# High occurrence of *Blastocystis *sp. subtype 3 in individuals referred to medical laboratories in Kermanshah, Iran 

**Published:** 2022

**Authors:** Bahman Maleki, Javid Sadraei, Abdolhossein Dalimi Asl, Majid Pirestani

**Affiliations:** *Department of Parasitology, Faculty of Medical Sciences, Tarbiat Modares University, Tehran, Iran*

**Keywords:** Blastocystis sp., Prevalence, Subtype, Kermanshah, STS PCR

## Abstract

**Aim:**

The current study investigated the prevalence and genotypes of *Blastocystis *sp. in individuals who referred to medical laboratories in Kermanshah, Iran.

**Background:**

*Blastocystis *sp*. *is a common intestinal protozoan found in humans and a wide range of animals, and it is involved in the development of gastrointestinal disorders.

**Methods:**

A total of 950 stool samples were examined using the standard formalin-ether concentration technique. All specimens were cultured in Robinson xenic medium. Subsequently, DNA extraction and PCR amplification of subtype specific sequence-tagged site (STS) were conducted.

**Results:**

Microscopic examination showed that 86 out of 950 samples (9.05%) were infected with *Blastocystis *sp. Subsequently, 33 of 86 positive samples were cultured and molecularly confirmed by conventional PCR, indicating six subtypes (ST1-ST6). Of note, ST3 (45.0%) was the predominant subtype, followed by ST1 (15.15%) and ST5 (12%).

**Conclusion:**

Based on the current findings, ST3 was the most frequent subtype among all positive samples. Having a better understanding of *Blastocystis *sp. subtype distribution and risk factors would lead to improved preventive measures.

## Introduction

*Blastocystis *sp. is a commonly found protozoan parasite of the intestinal lumen of insects and a wide range of vertebrates, including humans (1-3). *Blastocystis* sp*.* is transmitted through the fecal–oral route (infected food or water by cyst) (4, 5). Close contact with animals also represents a major predisposing factor for *Blastocystis *sp. transmission (6, 7). Despite its global prevalence, *Blastocystis *sp. infection has been frequently reported from developing nations. Several risk factors have been recognized in these areas, including inadequate access to safe drinking water, lack of sanitary infrastructures, and close contact with animals (8-10). 

Recent years have seen much controversy about the exact pathogenic mechanism of *Blastocystis *sp. Although many studies have determined this organism to be a commensal agent in healthy individuals (11-13), other investigations have isolated the parasite from patients with gastrointestinal disorders, such as acute or chronic diarrhea, abdominal pain, bloating, constipation, nausea, vomiting, and fatigue. Moreover, some studies have linked *Blastocystis *sp. infection to auto-immune diseases (i.e. irritable bowel syndrome (IBS) and urticaria allergic lesions) and anemia (14-17).

To diagnose *Blastocystis *sp. infection, microscopic examination, culture, and molecular methods are used. However, microscopy and culture methods are unable to accurately determine the parasite and its subtypes; hence, their use is limited. Culture medium, on the other hand, is more sensitive (18-20). Molecular methods have been extensively developed for the classification of* Blastocystis *sp. subtypes. Based on a recent molecular study on the small subunit ribosomal RNA (SSU-rRNA) or18S rRNA gene, *Blastocystis *sp. has been sorted into at least 26 subtypes (ST), and ST1-ST4 have been highly reported in humans (21, 22). Reportedly, specific STs may contribute to degrees of pathogenicity, although with conflicting results which demand further exploration. (9). 

**Table 1:** Seven pairs of sequence-tagged site primers (STS) along with two pairs of ST-specific primers and expected PCR product

Primers	PCR product size	GenBank accession Number	Sequences of forward (F) and Reverse (R) primers (5– 3)
SB83 Sub 1	351-bp	AF166086	F: GAAGGACTCTCTGACGATGA R: GTCCAAATGAAAGGCAGC
SB337 Sub 2	650-bp	AF166087	F: ATCAGCCTACAATCTCCTC R: ATCGCCACTTCTCCAAT
SB227 Sub 3	526-bp	AF166088	F: TAGGATTTGGTGTTTGGAGA R: TTAGAAGTGAAGGAGATGGAAG
SB336 Sub 4	338-bp	AF166091	F: GCATCCAGACTACTATCAACATT R: CCATTTTCAGACAACCACTTA
SB155 Sub 5	704-bp	AY048752	F: TGTTCTTGTGTCTTCTCAGCTC R: TTCTTTCACACTCCCGTCAT
SB340 Sub 6	317-bp	AY048751	F: GTGGGTAGAGGAAGGAAAACA R: AGAACAAGTCGATGAAGTGAGAT
SB332 Sub 7	487-bp	AY048750	F: GTCTTTCCCTGTCTATTCTGCA R: AATTCGGTCTGCTTCTTCTG
ST-specific Sub 8	1480-bp	AB107970	F: GAATGAAAACCAGTAGACTTAGTCTATTCG R: CTCTATTCCTTTTACAGACTAGAAAC
ST-specific Sub 9	1030-bp	AF408425	F: RAGAATGTCAAATTCTTGTAAAMTARTC R: CCCAGATACWMAAACGTATCCG

According to epidemiological studies, ST1-ST8 along with ST12 are of zoonotic concern, ST9 is exclusively anthroponotic, and ST10, ST11, and ST13-ST26 have been only identified in animals (22-24). Studies aimed at *Blastocystis *sp. subtyping would inevitably expand our knowledge on geographical distribution, host specificity, transmission routes, and pathogenicity of this commonplace parasite (25-27). In Kermanshah province, located in the west of Iran, no studies on genetic diversity of *Blastocystis *sp. have been conducted in human population. The current study aimed to assess the prevalence and genetic diversity of *Blastocystis *sp. isolates from humans in Kermanshah using the polymerase chain reaction (PCR) method and employing seven pairs of STS primers along with two pairs of ST-specific primers. 

## Methods

**Study area**

Kermanshah city (34°18′N, 47°3′E), the capital of Kermanshah province, is the largest city in the west of Iran with one million inhabitants. The city is located 1420 meters above sea level and possesses a mountain climate with an annual average rainfall of 500 millimeters (28). 

**Sample collection and parasitological examination**

The present cross-sectional study was conducted on a total number of 950 individuals who referred to the medical laboratories of Kermanshah University of Medical Sciences. The stool specimens were collected in a plastic container without fixative and sent to the parasitology laboratory for microscopic examination.

**Microscopic method**

All stool samples were concentrated using the formalin-ether technique and examined using Lugol’s iodine staining. Next, a cover slip was placed on the surface of each prepared sample, and the samples were examined by light microscopy at a magnification of 40× to detect *Blastocystis* sp*.* parasites.

**In-vitro culture of** ***Blastocystis*** **sp. isolates**

**Figure 1:**
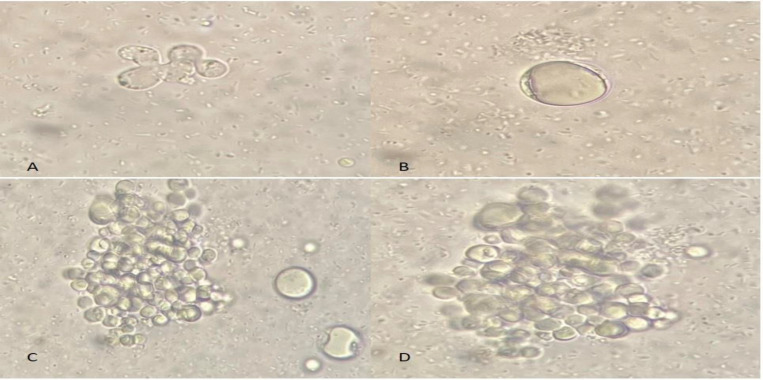
Amoeboid form of *Blastocystis* subtypes (A), Vacuolar form (B) Clumping of vacuolar form (C, D)

After microscopic examination, centrifugation (1200 rpm 5 min) was applied to remove debris from positive samples. Cultured parasites were then propagated in Robinson axenic culture medium at 37 °C (29) supplemented with erythromycin (100 μg/mL), penicillin (500 IU/mL), streptomycin (500 μg/mL), gentamicin (25 μg/mL), and amphotericin B (20 μg/mL) to inhibit the growth of bacterial and fungal organisms (30).

When the typical vacuolar or granular forms of *Blastocystis *sp. organisms were observed, they were sub-cultured in a new medium. The cultivated samples were examined every 48-72 h by iodine staining under a light microscope. Negative samples were checked for 5 consecutive days to confirm the negative result. 

**Extraction of Genomic DNA**

DNA extraction was performed on the pure *Blastocystis *sp. cultures, using QIAamp DNA Stool mini kit (cat. no. 51504) according to the manufacturer's instructions (30).

**Subtyping**

To determine the *Blastocystis *sp. subtype, we used seven pairs of STS (subtype specific sequence-tagged site) and two pairs of ST-specific primers (Table 1) (31, 32). The PCR reaction included 7.5 μL of 2X Taq DNA polymerase Mix Red-Mgcl2 (Ampliquon©, Denmark), 3 μL of extracted DNA, 1 μL of each primer (10 p mol), and 2.5 μL of sterile water. The PCR reaction was performed in a final volume of 15 μL.

PCR conditions for the nine primer pairs included an initial denaturation step at 94 ℃ for three min, followed by 35 cycles of including denaturation at 94 ℃ for 30 sec, annealing at 56 ℃ for 30 sec, extension at 72 ℃ for 30 sec, and a final extension step at 72 ℃ for five min. Subsequently, PCR products were loaded into wells of a plate with 1.5% agarose gel containing CinnaGen DNA safe stain and visualized using a UV transilluminator. The size of the amplicon was determined using a 100 bp DNA marker and represented the parasite subtype (Table 1). 

**Table 2:** Recognition subtypes of *Blastocystis* sp. among 33 culture samples by PCR method

Mono subtype infections				Mixed subtype infections							
Subtype	ST1	ST5	ST3	ST1/ST3 mix	ST1/ST5 mix	ST1/ST6 mix	ST1/ST5/ ST6 mix	ST5/ST2 mix	ST3/ST4 mix	ST3/ST5 mix	ST3/ST6 mix
Positive number (%)	5(15.15%)	4(12%)	15(45%)	2(6%)	1(3%)	1(3%)	1(3%)	1(3%)	1(3%)	1(3%)	1(3%)
Total mono infections 24(73%)				Total mixed infections 9 (27%)							

**Table 3:** Number of single and mixed subtype

subtype	ST1	ST5	ST3	ST6	ST4	ST2	Total
Mono subtype infections	5	4	15	0	0	0	24
Mixed subtype infections	4	4	4	3	1	1	17
Total	9	8	19	3	1	1	41

## Results

The current results indicated that only 86 out of 950 (9.05%) fecal samples were infected with *Blastocystis* sp. parasites microscopically. Subsequently, the parasites were grown in 33 cultivated stool samples and further subtyped using the PCR method (Figure 1). All samples originating from positive cultures were further subtyped using the PCR method (Table 2). Molecular tests revealed a total of six different subtypes (ST1, ST2, ST3, ST4, ST5, and ST6) (Table 2). 

In single infections, ST3 was the most highly prevalent subtype (n=15, 45%) followed by ST1 (n=5, 15%) and ST5 (n=4, 12%) (Table 2). Mixed infections, observed in nine positive samples, increased the number of isolated subtypes, so that nine ST1, eight ST5, and eighteen ST3 were detected (Table 3). The predominant subtypes reported in mixed infections were ST1/ST3 (2/9; 22.2%), and other subtypes were detected as mixed, comprising ST1/ST5, ST1/ST6, ST1/ST5/ ST6, ST5/ST2, ST3/ST4, ST3/ST5, and ST3/ST6(Tables 2 and 3).

## Discussion

The parasites of the genus *Blastocystis *sp. demonstrate a high rate of genetic diversity and varied host specificity worldwide (9). Epidemiological studies have reported different prevalence rates in examined human populations, which could be attributed to demographic settings such as cultural habitats, sanitation levels, the socioeconomic status of the affected people, rural living areas, exposure to animal reservoirs, and diagnostic method (8, 9). Before 2013, molecular analysis of SSU rRNA locus using STS primers revealed 17 subtypes, while ST18-ST26 were proposed after 2013. Later in 2020, Stensvold et al. rejected STs 18-20 and ST22 according to the guidelines of *Blastocystis *sp. subtyping and recommended keeping ST21 and STs 23-26 until further examination (9, 24). 

In the present study, *Blastocystis *sp. was detected in 86 (9.05%) of the 950 stool specimens examined by routine diagnostic method (formalin-ether technique). To prevent misdiagnosis due to microscopy, eliminate stool inhibitors, and provide pure parasites, the culture method was preferred (33, 34). In addition, it’s possible that some *Blastocystis *sp. subtypes do not grow or grow slowly in axenic culture (18). According to Robert et al., the PCR method is a highly sensitive method (94%) for detecting *Blastocystis *sp.*,* compared with microscopy (48%) (18).

In the present study, 33 microscopy-positive samples were grown on Robinson culture medium, all of which were subtyped using the PCR method.

Previously, several studies employed STS primers, ITS sequencing, PCR-RFLP, and Real-time PCR to identify *Blastocystis *sp*.* subtypes in Iran (6, 35, 36).

The current findings identified ST1(15.15%), ST5 (12%), ST3 (45%), ST6 (9%), ST4 (3%), and ST2 (3%). Moreover, ST3, ST1, and ST5 as the most prevalent subtypes, respectively, were isolated in both single and mixed infections, whereas ST6, ST4, and ST2 were found only in mixed infections. The most frequent subtype in this study was ST3 (45%), which is consistent with Iranian studies conducted in Ahvaz, Sanandaj, Tehran, west Azerbaijan, and Lorestan as well as investigations from other countries, including Turkey (Asia), Thailand (Asia), Indonesia (Asia), Italy (Europe), China (Asia), Australia (Oceania), and Egypt (Africa) (36-48).

Although ST3 demonstrates strict host-specificity to humans, it has rarely been isolated from primates, pigs, dogs, cattle, or rodents (21, 49, 50).

Reportedly, symptomatic and asymptomatic people are prone to this subtype, while other studies have denied an association between *Blastocystis *sp. and gastrointestinal symptoms (26, 37, 51-53). In this sense, El Safadi et al. (2014) showed ST3 to be a seemingly frequent subtype in the symptomatic group (54).

ST1 has been said to have low host specificity and has been isolated from humans as well s a range of animals including monkeys, chickens, cattle, pigs, dogs, and non-human primates (55, 56). In contrast to the current findings, ST1 was markedly prevalent in other parts of Iran (Qazvin and Hamadan), Libya (Africa), Thailand (Asia), Brazil and Colombia (Latin America) (9, 55, 57-60). These observations highlight large-scale inter-human transmission (9, 61). 

Zoonotic transmission of ST1 in the Kermanshah population is a possibility, because some families live in the suburbs near farm animals. Nevertheless, no investigation has been done on animal hosts in this area. Salehi et al. (2020) conducted a study on the prevalence and distribution of *Blastocystis *sp*.* subtypes in non-human hosts (poultry, sheep, and cattle) in the southwest region of Iran and reported a prevalence rate of 29.1% in animal hosts; ST1 was also confirmed in two cattle samples. Another study in Khorramabad, in western Iran, ST1 was not found in cattle. In a recently published paper, Rahimi et al. detected *Blastocystis *sp*.* in cattle, sheep, and chicken samples and confirmed ST1 in cattle (6, 7, 27).

Alfellani et al. (2013) emphasized that animal infection with a particular subtype may not reasonably justify its presence in humans (9). 

Regarding the isolation of ST5, in four single and four mixed specimens, zoonotic transmission is a possibility, because cattle and pig are dominant reservoir animals for this subtype. Cattle husbandry around Kermanshah may be considered a reason for the presence of a zoonotic cycle. Previously, this subtype was reported from human and livestock in the south, west, and southwest of Iran (7, 27, 36, 62-64). 

*Blastocystis* sp. ST2 is a major subtype globally, and has been found to be the most frequent subtype in some studies in Iran (Tehran and Shiraz) and other parts of the world, such as Turkey, Spain, and Bolivia (62, 65-69, 59, 70). In contrast, the current study detected ST2 only in a sample with mixed infections.

Additionally, ST4, which had the lowest prevalence in the current study (3%), was the most common subtype in southwestern Iran, France, Spain, and Nepal (63, 71-74).

Although rodents are reservoir hosts for ST4 and are found all over the world, this subtype is most common in Europe. This may be due to the lack of information in many Asian countries and Africa. In an Iranian study by Mohammadpour et al. (2020), ST4 was isolated from cats and rats in Fars province, in southern Iran (75).

Birds are commonly infected with ST6, which has also been documented in livestock and humans (23). The current results showed the presence of ST6 in Kermanshah people, as observed in three mixed samples. Previously, this subtype was reported in humans, poultry, and cattle in the southwest, west, and south of Iran (24, 49, 55, 64). Of note, ST7, ST8, and ST9 were not identified in human population of Kermanshah.

Overall, several *Blastocystis *sp*.* subtypes can be found in human fecal samples simultaneously, and such mixed infections may result from various sources of infection (40). According to different studies, the prevalence of mixed infections ranges 1.1–14.6%, whereas herein, a higher prevalence was found (27%) (57). Moreover, the current study characterized ST1/ST3 and ST5 in mixed infections from Kermanshah, while other Iranian studies have reported ST1 and ST3 in them (57, 64, 76). A major finding of the current study was that some rare subtypes were distinguished in mixed infections, comparable to other studies across the country (26, 36, 57). In total, the evaluation of mixed infections and relevant subtypes is a cumbersome procedure and requires pure parasite cultures and molecular identification using subtyping methods (40).

To the best of the authors’ knowledge, this is the first molecular study to investigate the distribution of human *Blastocystis *sp*.* subtypes in Kermanshah, Iran. Evidence of the molecular epidemiology of *Blastocystis *sp*.* in all parts of Iran is insufficient. In this study, ST3 was the most common subtype, followed by ST1, ST5, ST6, and ST4. Further studies are needed to identify *Blastocystis *sp*.* subtypes in different hosts. Recognition of *Blastocystis *sp*.* subtype distribution may help our understanding of the associated risk factors and transmission routes. Overall, the findings represented here indicate the possibility of zoonotic transmission of *Blastocystis *sp. in Kermanshah city.

## Conflict of interests

The authors declare that they have no conflict of interest.
